# Effect of Pre-Heating on the Monomer Elution and Porosity of Conventional and Bulk-Fill Resin-Based Dental Composites

**DOI:** 10.3390/ijms232416188

**Published:** 2022-12-19

**Authors:** Erika Dunavári, Gergely Berta, Tamás Kiss, József Szalma, Márk Fráter, Katalin Böddi, Edina Lempel

**Affiliations:** 1 Department of Restorative Dentistry and Periodontology, University of Pécs Medical School, Tüzér Street 1, 7624 Pécs, Hungary; 2 Department of Medical Biology and Central Electron Microscope Laboratory, University of Pécs Medical School, Szigeti Street 12, 7624 Pécs, Hungary; 3 Szentágothai Research Centre, University of Pécs, Ifjúság Street 20, 7624 Pécs, Hungary; 4 Department of Oral and Maxillofacial Surgery, University of Pécs Medical School, Tüzér Street 1, 7624 Pécs, Hungary; 5 Department of Operative and Esthetic Dentistry, Faculty of Dentistry, University of Szeged, Tisza Lajos Street 64, 6720 Szeged, Hungary; 6 Department of Biochemistry and Medical Chemistry, University of Pécs Medical School, Szigeti Street 12, 7624 Pécs, Hungary

**Keywords:** resin composite, bulk fill, pre-heating, monomer elution, porosity

## Abstract

The pre-heating of dental resin-based composites (RBCs) improves adaptability to cavity walls, reducing microleakages. However, the rapid cooling of the pre-heated RBC may change the polymerization kinetics, and thus the final network configuration of the RBC. It is well known that unreacted monomers remaining in the set RBC can leach into the oral cavity. However, it is still not clear how the pre-heating and cooling of RBCs alter monomer elution (ME). Thus, the purpose was to determine the ME from room-temperature and pre-heated RBCs, in addition to determining the closed porosity (CP) volume. Bulk-filled RBCs and layered conventional RBC samples were prepared. The pre-polymerization temperature was set at 24 °C and 55/65 °C. The ME from RBC samples was assessed with high-performance liquid chromatography using standard monomers. CP was measured with micro-computed tomography. ME decreased significantly from bulk fills and increased from layered samples as a result of pre-heating. Pre-heating was unfavorable in terms of CP in most RBCs. Based on the effect size analysis, ME and CP were greatly influenced by both material composition, pre-polymerization temperature, and their interaction. While the pre-heating of high-viscosity bulk-fill RBCs is advantageous from a clinical aspect regarding biocompatibility, it increases CP, which is undesirable from a mechanical point of view.

## 1. Introduction

As one of the most common dental restorative materials, resin-based composites (RBCs) have been widely and effectively used in clinical practice [1]. Their gradual improvement in formulation, properties, and esthetics has made them popular among dentists [2]. In order to achieve durable and successful direct restoration, the most important factors include the mechanical properties, degree of conversion (DC), polymerization stress, handling characteristics, and marginal adaptation [3]. High viscosity and stickiness impair the handling of the RBC, resulting in deficient adaptation to the cavity walls and margins and potentially increasing void incorporation [4,5]. Flowable RBCs present significantly lower viscosity, which can improve the adaptation through the increased wettability of the walls [4]. Viscosity, among other things, is strongly dependent on the composition of the resin matrix, filler size, distribution, and fraction [6]. A lower filler load is reflected in inferior functional wear and physical properties, and therefore are drawbacks of flowable RBCs [7]. However, a significant reduction in viscosity can be achieved by the pre-heating of condensable RBCs without compromising the mechanical properties [8]. At elevated temperatures, thermal vibration allows the monomers to slide by each other more easily, leading to decreased internal friction [9]. The increased flow of pre-heated condensable RBCs improves the adaptation of the uncured material to the cavity walls, potentially reducing microleakages [10]. In addition to good adaptation, other advantages of pre-heated RBCs, such as a higher DC, increased surface hardness, and compressive and diametral tensile strength, have made them popular among clinicians [10,11]. In addition, flexural strength is unaffected, while polymerization shrinkage is adversely influenced by pre-heating [12].

Since an inverse correlation was detected between the DC and unreacted monomer elution from RBCs by several investigations, the question arises as to how pre-heating affects the biocompatibility of RBCs [13,14]. The DC expresses the extent of polymerization. A higher DC corresponds to increased microhardness, flexural strength and modulus, fracture resistance, tensile strength, dimensional and color stability, and decreased solubility and monomer elution [15,16,17,18]. RBC polymers with similar DCs may exhibit distinct cross-link densities due to the differences in polymerization kinetics [10,19]. The process of monomer-to-polymer conversion and the properties of the set polymer network are influenced by the polymerization temperature [10]. Pre-heating transfers heat energy to the system, which changes the kinetics due to the reduction in viscosity and the promotion of reactive radical mobility and collision frequency, which results in higher monomer-to-polymer conversion and delayed autodeceleration [20]. This may alter the network configuration and may influence the arrangement of polymerized, pendant, and free monomer molecules [21,22]. Several studies concluded that rapid cooling during removal from the heating device may compromise the monomer-to-polymer conversion depending on the type of RBC [23,24,25]. In a recent study, pre-heating was not shown to influence the DC of a newly introduced thermoviscous bulk fill; however, a decreased DC at the bottom surface of a contemporary bulk-fill RBC was shown [26]. As a consequence of incomplete polymerization, the unreacted and also pendant monomers can reduce the physical properties of the RBCs, and their detection plays an important role in the evaluation of RBC biocompatibility [27].

The amounts of leachable monomers could be higher in the presence of oxygen-containing pores and voids since the oxygen-inhibited layer is similar to the unpolymerized RBC and may potentially release unreacted monomers [28]. Upon investigating the impact of different insertion techniques on internal pore and void formation, Demirel et al. found that the application of a thermoviscous bulk-fill RBC with the utilization of the pre-heating technique showed the lowest void percentage [29].

As the result of the above-referred studies, pre-heating and the consequential rapid cooling may differently alter the polymerization kinetics of resin composites depending on their composition and manipulation method. Thus, the final network configuration and the arrangement of the connected and unconnected monomer units could be different. In addition to the setting kinetics, the application technique may also influence pore formation, leading to a change in the amount of leachable unreacted monomers. Although the release of unreacted monomers from room-temperature conventional or bulk-fill RBCs has been extensively investigated, there is no available literature about how pre-heating may change monomer release and porosity, which are important factors with regard to biocompatibility.

Therefore, the purpose of the present study was to investigate the unreacted monomer release from different types of restorative RBCs as a result of pre-heating, using reversed-phase high-performance liquid chromatography (RP-HPLC). A further aim was to evaluate the effect of pre-heating on the closed porosity of RBCs with the help of micro-computed tomography (micro-CT) measurements. The null hypotheses were: (1) pre-heating has no effect on the porosity of RBCs, and (2) pre-cure temperature does not affect the amount of released unreacted monomers.

## 2. Results

The maximum radiant exitance of the LED LCU measured by the radiometer was 1250 ± 15 mW/cm^2^. The delivered radiant exposure was 25 ± 3 J/cm^2^.

### 2.1. Micro-Computed Tomography Measurements

**Figure 1:**
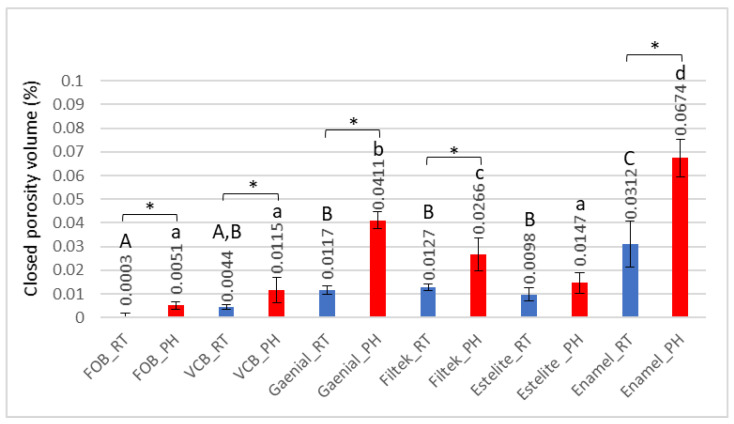
Closed-porosity volume (%) of the room temperature and pre-heated samples analyzed with micro-computed tomography. Different letters denote statistically significant differences among the materials analyzed by one-way analysis of variance (ANOVA) and Tukey’s post hoc test. The * mark indicates statistically significant differences between the room-temperature (RT) and pre-heated (PH) groups according to the independent two-tailed *t*-test.

According to the 3D evaluation, the volume of internal voids relative to the total volume of the RBC sample was higher in pre-heated samples compared to the room-temperature samples for each material (Figure 1). The differences found to be significant according to the independent *t*-test were FOB: t(4) = −6.26; *p* < 0.001; VCB: t(4) = −2.99; *p* < 0.02; FZ: t(4) = −4.46; *p* = 0.002; GP: t(4) = −16.37; *p* < 0.001; EP: t(4) = −6.3; *p* < 0.001, except ESQ t(4) = −2.09; *p* = 0.07.

Among the investigated materials, the lowest porosity was detected in the bulk-fill samples (FOB and VCB), while the highest values were found in EP, especially in its pre-heated form. The order of measured porosities among RBCs was: FOB < VCB < ESQ < FZ < GP < EP.

The general linear model revealed that both the *Material* (*p* = 0.001) and *Temperature* (*p* = 0.001) factors have a significant effect on closed porosity and the effect size was considered to be large (*Material* ƞp^2^ = 0.38; *Temperature* ƞp^2^ = 0.18).

### 2.2. Reversed-Phase High-Performance Liquid Chromatography Measurements

Bisphenol A-glycidyl methacrylate (BisGMA), urethane-dimethacrylate (UDMA), trietylene-glycol-dimethacrylate (TEGDMA), 1,12-dodecanediol-dimethacrylate (DDMA), and tricyclodecane-dimethanol-dimethacrylate (TCDDD) standard monomers were selected to detect the elution of these monomers from the investigated bulk-fill and layered contemporary RBCs. In addition to the monomers specified by the manufacturers, other methacrylates were also detected from FOB (BisGMA), VCB (TEGDMA), and EP (DDMA) RBCs. Although TCDDD is a component of GP, the free monomer was not released in a detectable amount.

**Figure 2:**
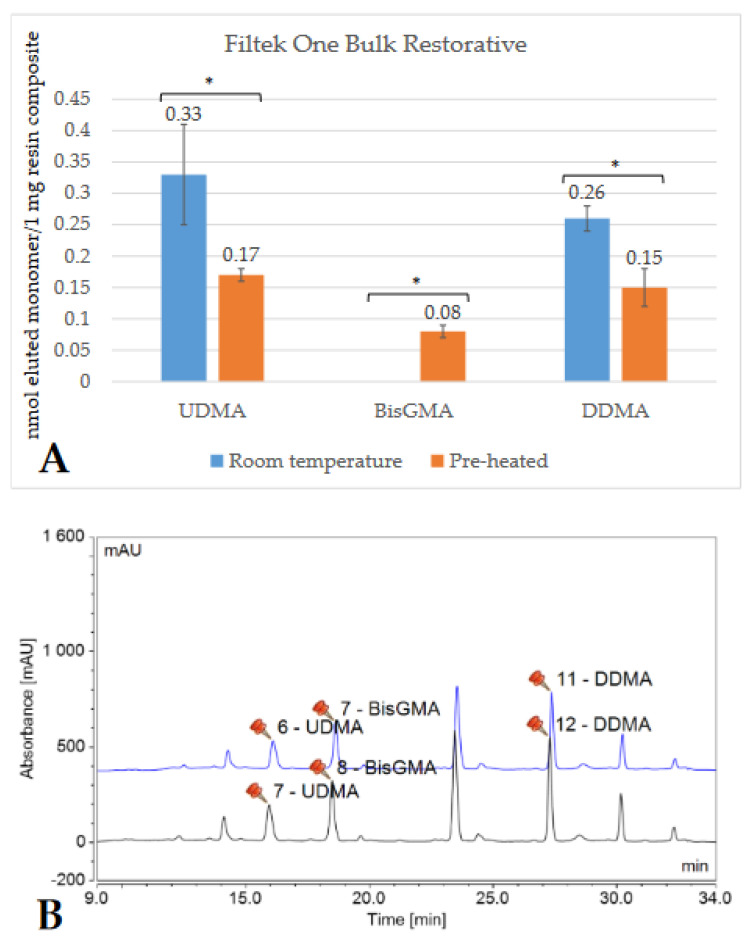
Comparison (**A**) of the amount of eluted monomers from room-temperature (blue bars) and pre-heated (orange bars) Filtek One Bulk Restorative samples. The * mark indicates statistically significant differences between the groups according to the independent two-tailed *t*-test. The chromatogram (**B**) shows the detected monomers eluting from room-temperature (black chromatogram) and pre-heated (blue chromatogram) Filtek One Bulk Restorative (FOB) and their retention times based on the standard monomers, evaluated at a 205 nm wavelength. (Abbreviations: UDMA, urethane-dimethacrylate; BisGMA, bisphenol A-glycidyl methacrylate; DDMA, 1,12-dodecanediol-dimethacrylate; mAU, milli-Absorbance Units).

**Figure 3:**
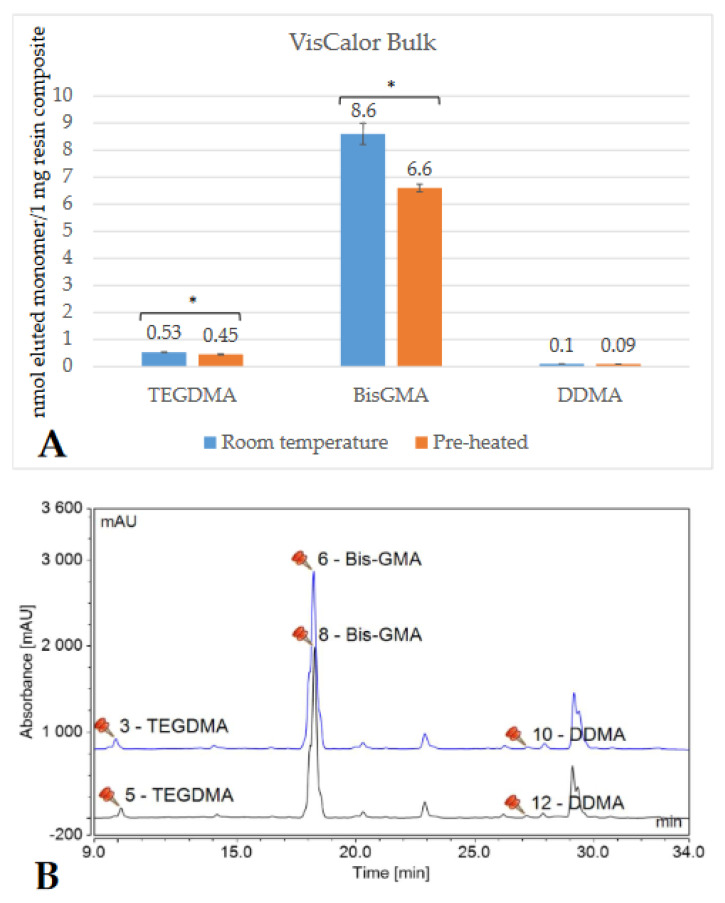
Comparison (**A**) of the amount of eluted monomers from room-temperature (blue bars) and pre-heated (orange bars) VisCalor Bulk samples. The * mark indicates statistically significant differences between the groups according to the independent two-tailed *t*-tests. The chromatogram (**B**) shows the detected monomers eluting from room-temperature (black chromatogram) and pre-heated (blue chromatogram) VisCalor Bulk and their retention time based on the standard monomers, evaluated at a 205 nm wavelength. (Abbreviations: BisGMA, bisphenol A-glycidyl methacrylate; TEGDMA, trietylene-glycol-dimethacrylate; DDMA, 1,12-dodecanediol-dimethacrylate; mAU, milli-Absorbance Units).

**Figure 4:**
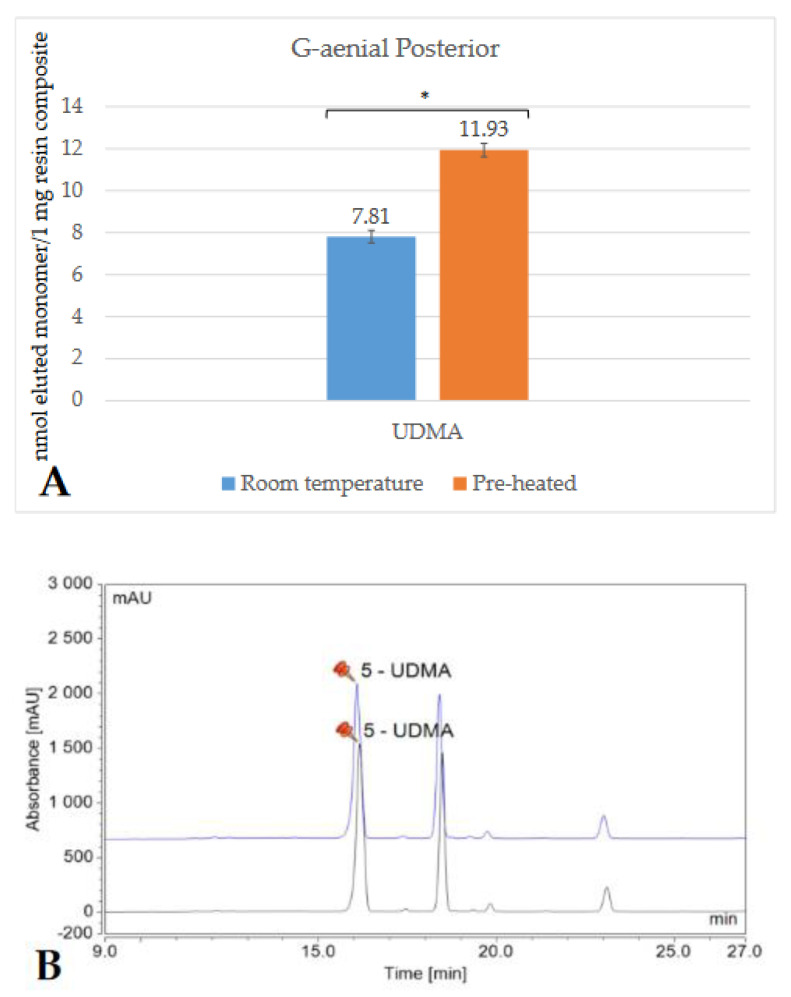
Comparison (**A**) of the amount of eluted monomers from room-temperature (blue bar) and pre-heated (orange bar) G-aenial Posterior samples. The * mark indicates statistically significant differences between the groups according to the independent two-tailed *t*-test. The chromatogram (**B**) shows the detected monomers eluting from room-temperature (black chromatogram) and pre-heated (blue chromatogram) G-aenial Posterior and their retention times based on the standard monomers, evaluated at a 205 nm wavelength. (Abbreviations: UDMA, urethane-dimethacrylate; mAU, milli-Absorbance Units).

**Figure 5:**
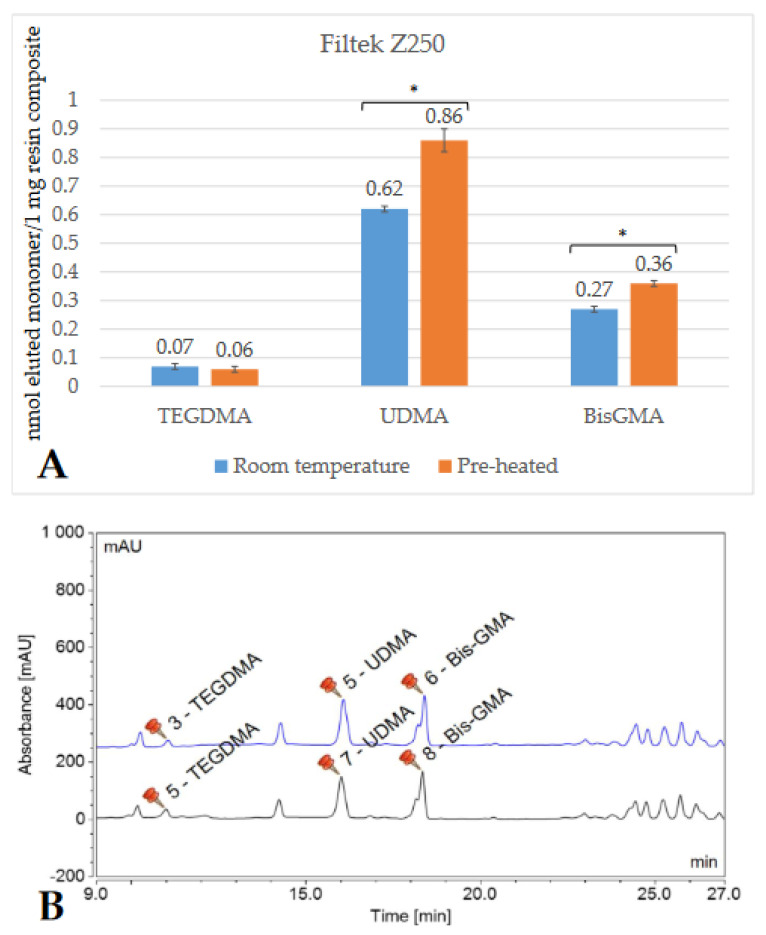
Comparison (**A**) of the amount of eluted monomers from room-temperature (blue bars) and pre-heated (orange bars) Filtek Z250 samples. The * mark indicates statistically significant differences between the groups according to the independent two-tailed *t*-test. The chromatogram (**B**) shows the detected monomers eluting from room-temperature (black chromatogram) and pre-heated (black chromatogram) Filtek Z250 and their retention times based on the standard monomers, evaluated at a 205 nm wavelength. (Abbreviations: TEGDMA, trietylene-glycol-dimethacrylate; UDMA, urethane-dimethacrylate; BisGMA, bisphenol A-glycidyl methacrylate; mAU, milli-Absorbance Units).

**Figure 6:**
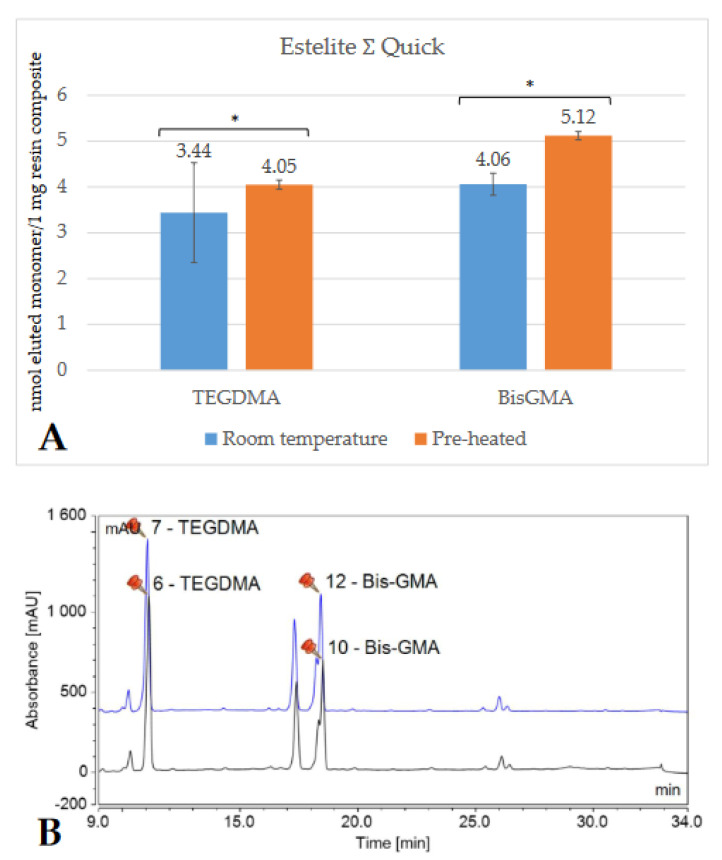
Comparison (**A**) of the amount of eluted monomers from room-temperature (blue bars) and pre-heated (orange bars) Estelite ƩQuick samples. The * mark indicates statistically significant differences between the groups according to the independent two-tailed *t*-test. The chromatogram (**B**) shows the detected monomers eluting from room-temperature (black chromatogram) and pre-heated (blue chromatogram) Estelite ƩQuick and their retention times based on the standard monomers, evaluated at a 205 nm wavelength. (Abbreviations: TEGDMA, trietylene-glycol-dimethacrylate; BisGMA, bisphenol A-glycidyl methacrylate; mAU, milli-Absorbance Units).

**Figure 7:**
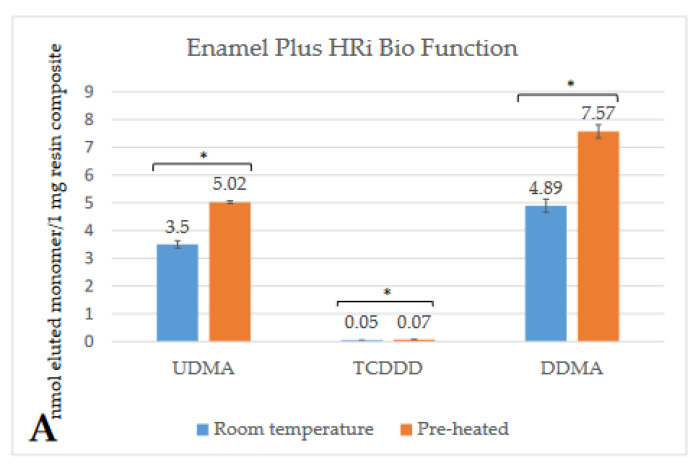

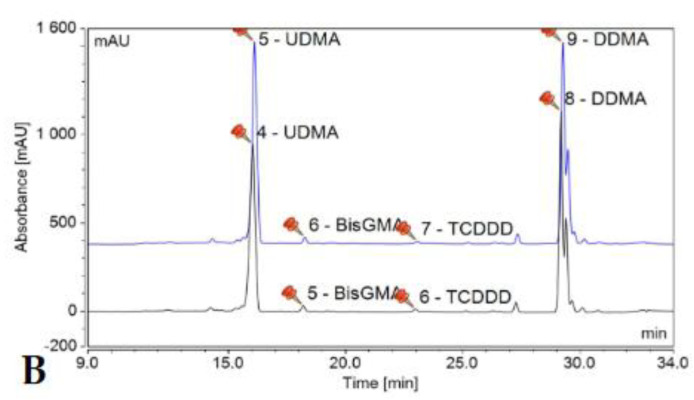
Comparison (**A**) of the amount of eluted monomers from room-temperature (blue bars) and pre-heated (orange bars) Enamel Plus HRi Bio Function samples. The * mark indicates statistically significant differences between the groups according to the independent two-tailed *t*-test. The chromatogram (**B**) shows the detected monomers eluting from room-temperature (black chromatogram) and pre-heated (blue chromatogram) Enamel Plus HRi Bio Function and their retention times based on the standard monomers, evaluated at a 205 nm wavelength. (Abbreviations: UDMA, urethane-dimethacrylate; DDMA, 1,12-dodecanediol-dimethacrylate; TCDDD, tricyclodecane-dimethanol-dimethacrylate; mAU, milli-Absorbance Units).

Depending on the brand, and thus the composition of the RBC, the detected monomer elution varied extensively. The highest amount of monomer was released from GP (UDMA), followed by VCB (BisGMA), then EP (DDMA, UDMA), and ESQ (BisGMA, TEGDMA). Less than 1 nmol/1 mg RBC monomer elution was detected from both the conventional (FZ) and bulk-fill (FOB) Filtek RBCs. Figure 2, Figure 3, Figure 4, Figure 5, Figure 6 and Figure 7 show the chromatograms and the amount of identified eluted monomers from the investigated room temperature and pre-heated RBCs. Several chromatograms show peaks of eluted, but not identified substances besides the monomers identified as standard dimethacrylates.

The evaluated monomers from both the bulk-fill FOB and VCB showed a significantly greater degree of elution when applied at room temperature compared to the pre-heated samples. The monomer elution from the FOB_55 decreased by ~50%, while the elution from VCB_65 was reduced by 15–20%. However, the opposite tendency was observed in the case of the layered contemporary RBCs. Their monomer release was significantly higher in the pre-heated groups (in GP_55, increased by 35%; in FZ_55 by 25–30%; in ESQ _55 by 15–20%; and in EP_55 by 30–35%) (Table 1).

The general linear model showed a statistically significant effect for both the *Material* and *Temperature* factors and also for their interaction on BisGMA, UDMA, TEGDMA, and DDMA elution. The partial eta-squared value was considered to be large for both factors and their interaction, as well (Table 2).

The TCDDD monomer was not included in the effect size analysis, since its release was only detected from EP.

## 3. Discussion

In this in vitro study, the volume of the internal pores and the elution of the unreacted monomers of room-temperature and pre-heated conventional layered and bulk-fill dental resin composites were assessed using micro-computed tomography and high-performance liquid chromatography.

It was assumed that (1) pre-heating has no effect on the porosity of RBCs and that (2) the pre-cure temperature does not affect the amounts of unreacted monomers released. However, both hypotheses should be rejected, since the pre-heating of the investigated RBCs had a significant impact on internal pore formation as well as on monomer elution.

The accuracy of three-dimensional, high-resolution micro-CT allowed the detection of small voids and air incorporation within the RBCs [30]. Although submicron pores are already present in the material delivered by the manufacturer [31], further air bubbles are incorporated into the restoration during the clinical manipulation [32]. Porosity is an undesirable property of the RBC, which can significantly decrease the strength, increase water solubility and microleakages, and thus compromise the success of the restoration [33,34].

Porosity correlates with layer thickness, placement technique, and operator skill [35,36]. Our findings showed that the two bulk placements (FOB and VCB) resulted in less pore formation compared to the ones that were layered (GP, FZ, ESQ, and EP); however, the values do not differ in a statistically significant manner in every case.

Our findings are consistent with other studies that evaluated different techniques of posterior RBC placement [35,37]. They found a high rate of air incorporation in the examined samples, but a higher incidence of pores was found in the samples that were layered in thicker increments [31].

A more pronounced effect was found on pore formation when the RBCs were pre-heated. Compared to the room temperature samples, significantly higher closed porosity values were measured as a result of pre-heating in all tested RBCs, except ESQ. Pre-heating reduces the viscosity of RBC, which helps material handling as it flows into the cavity. Although several studies showed that the use of high-viscosity RBCs increased the risk of air incorporation, it was also detected that the use of flowable RBCs cannot eliminate the risk of void formation [35,38]. However, Balthazard et al., through their investigation into the porosity of RBCs, concluded, that the less viscous the material was, the greater its porosity, regardless of the handling conditions [39]. Highly viscous RBCs need a higher condensing force to adapt the material to the walls, which may squeeze out the air bubbles from the material [39].

Furthermore, the vaporization of monomers also resulted in increased porosity. The elevation of the pre-cure temperature increases the vaporization of the organic resin components, especially the low-molecular-weight monomers [34].

Although porosity strongly depends on the consistency and handling conditions, it is a multifactorial phenomenon and is also influenced by the polymerization of the material. The curing protocol and the resin matrix composition have an impact on the polymer network architecture, and its heterogeneity may lead to increased porosity [40]. In contrast to studies reporting the improved monomer conversion of pre-heated RBCs under isothermal conditions, in a clinical setting, the RBC shows rapid cooling after removal from the warming device [41,42]. In a recent study, it was detected that the temperature of the pre-heated RBC decreased by ~26 °C immediately after removal from the heating device regardless of the pre-heating temperature. Further decreases by 1–2.6 °C were measured during RBC application into the mold [26]. The equilibration of the pre-heated RBCs and the ambient temperature resulted in faster cooling of the warmer RBCs, which may compromise the polymerization kinetics [23]. The rapid temperature decrease results in excess heat loss, which may deprive energy from the system, hinder the exothermic reaction, and prevent a sufficient increase in polymerization reactivity. Since the exothermic reaction during RBC polymerization reflects the extent of monomer-to-polymer conversion [10,43], the hindered propagation with reduced exothermic reaction can lead to a decreased DC, as was measured by Kincses et al. [26]. Moreover, the rapid cooling of the pre-heated RBC before polymerization may contribute to the structural heterogeneity of the polymer network [44]. A rapid decrease in temperature may lead to early vitrification, which restricts molecular mobility and hinders the diffusion of air bubbles which will become trapped in the material [40], resulting in higher porosity detected by micro-CT.

Among the above-mentioned phenomena, oxygen diffusion, as a side effect of elevated RBC temperatures, may play a significant role in the increased porosity of pre-heated RBCs [45]. As the temperature increases, the decrease in viscosity promotes oxygen penetration into the RBC. Oxygen also reduces the extent of conversion by scavenging on free radicals, resulting in less-reactive peroxy radicals and/or the quenching of the excited state of the initiator [45].

Insufficient polymerization decreases the degree of conversion and, as a consequence, might increase the released unreacted components from the RBC [15,17]. Unreacted monomers are not only the result of insufficient conversion. They are also present on the inner surface of air inclusions—related to porosity formation—due to oxygen inhibition [34,46]. The above issues may compromise the biocompatibility of RBCs and create favorable conditions for the proliferation of microorganisms [47]. The solubility of RBCs, furthermore, can accelerate degradation, which adversely affects the physical properties of the material [14].

RP-HPLC was used in this study to detect the released monomers, as it allows the accurate quantitative detection of monomers of interest. The analysis of selected unreacted dimethacrylates (BisGMA, UDMA, TEGDMA, DDMA, TCDDD) cannot provide an absolute measure of the quality of released components, as the chromatograms demonstrated other unidentified eluted ingredients. This can be considered a limitation of this study. Furthermore, the literature regarding monomer release from pre-heated RBCs is limited; hence, the discussion of this issue and subsequent comparisons to other results are also restricted. The amounts of eluted monomers were given in the nanomolar range, which was then converted to elution from 1 mg RBC to exclude the differences that arise from distinct weights of RBC samples. In clinical situations, the local concentrations of leached monomers can be in the millimolar range, which is considered to be high enough to induce a variety of adverse biological effects [22].

A 75 vol% ethanol/water medium was applied to extract the unreacted monomers as it is considered to be a good food simulator, and therefore clinically relevant according to the US Food and Drug Administration (FDA) [48].

Regarding the monomer elution in this study, distinctive results were found in high-viscosity conventional and bulk-fill RBCs. The former released 15–35% more monomer from its pre-heated form, meanwhile the monomer elution from bulk fills was reduced by 15–20% (VCB_65) and 50% (FOB_55) when the RBC was warmed.

Bulk-fill materials have higher volume, which can keep the increased temperature inside the material for a relatively prolonged period [49]. This can store enough energy in the polymerizing system for a higher DC even if the system temperature decreases rapidly. Additionally, high filler content can also contribute to the thermal storing capacity and may delay autodeceleration, thereby prolonging the vitrification of the polymer mass [23]. The absolute filler content and thus the filler/matrix interface is also higher in thicker restoratives. A possible role for the silane interface was suggested by Pluddemann, according to which the coupling agent can create dynamic thermal equilibrium between the organic matrix and inorganic filler, which results in a greater exothermic reaction during polymerization [50]. The results of recent studies, however, only partially confirm the above speculation regarding the DC of highly filled, pre-heated bulk-fill RBCs [23,26,42]. These findings support the strong effect of RBC composition on the results, as was also detected in our study [25]. Although the DC and monomer elution are inversely related [51], Kincses et al. detected contradictory results, as such—that pre-heating not only decreased the DC of FOB but also the monomer elution [26]. Chaharom et al. investigated the monomer release of pre-heated bulk-fill RBCs and detected slightly lower, although not significantly, elution from the pre-heated samples compared to the room-temperature specimens [52]. According to our results, pre-heating significantly reduced the unreacted monomer release from both bulk-fill RBCs. On the contrary, the monomer release was increased when the conventional layered RBCs were pre-heated. A possible explanation may be that the above-mentioned rapid cooling and, additionally, the cold-condensing instruments deplete more energy from the thinner (2 mm) layers. This may accelerate the vitrification and, with that, the termination of polymerization, leading to a decreased DC, a more heterogeneous network formation, and, thus, a higher amount of unreacted monomers. In contrast, Deb et al. found that increasing the pre-cure temperature to 60 °C in conventional RBCs caused a significant increase in the DC [53]. However, as was mentioned above, monomer release from RBC does not solely depend on the DC and the nature of the monomers but also may be related to the chemical structure of the polymer network. In polymerized structures, monomers stuck in micro-porosities are more susceptible to elution during water sorption, and heterogeneous materials have a higher volume of micro-pores [54].

In general, regardless of pre-cure temperature, a high amount of monomer was released from GP, followed by VCB, then EP, and ESQ; meanwhile, the monomer elution from FOB and FZ was fairly small. Despite the reduced monomer elution seen with the application of pre-heating, the absolute amount of BisGMA was still the highest from VCB (6.6 nmol/1 mg RBC) compared to all the investigated BisGMA-based RBCs. VCB is a BisGMA/aliphatic dimethacrylate-based thermoviscous RBC, according to the technical product profile. Following HPLC measurements, small amounts of TEGDMA and DDMA were detected as eluted aliphatic dimethacrylates. Strong intermolecular hydrogen bonds make BisGMA the most viscous molecule among resin matrix monomers. The strong bonding can decrease the reactivity and mobility of BisGMA during polymerization, which may lead to a higher amount of unconverted monomers [55]. On the other hand, the 75% ethanol/water used as an extraction medium showed easy penetration, especially in BisGMA-based RBCs. The resulting resin-softening expands the spaces within the polymer, creating soluble units [56]. In support of the above hypotheses, other studies also demonstrated elevated BisGMA, TEGDMA, and UDMA levels in the alcohol-based medium [13,57]. Like VCB, ESQ is a BisGMA/TEGDMA-based system, and the detected elution was high not only for BisGMA but also for TEGDMA. It can be assumed that the higher TEGDMA ratio resulted in higher elution compared to VCB. Additionally, TEGDMA is a low-molecular-weight monomer with higher mobility, which allows for a higher and faster rate of elution [58]. The UDMA-based GP and EP released high amounts of UDMA and diluting monomers, such as TEGDMA, DDMA, or TCDDD as well.

Although FOB is also a UDMA-based RBC and FZ is a BisGMA/UDMA/BisEMA-based restorative material, the elution of different monomers from both RBCs was 10 to 70-fold lower compared to the other RBCs tested, regardless of pre-curing temperature. One would assume that the similarly very low monomer dissolution is caused by the identical composition of the two RBCs. However, neither the organic matrix nor the filler content is the same. Moreover, FOB is a bulk fill, while FZ is a conventional RBC from the same company. In addition to UDMA, AUDMA, and DDMA, FOB also released BisGMA, although this monomer is not an officially listed constituent of this RBC. FOB contains a so-called addition fragmentation monomer (AFM) which contains a complementary internal double bond with a β-quaternary carbon center functional group. This provides living polymerization where the active center effectively diffuses throughout the network, simultaneously creating free radicals that initiate a new propagating radical and enable bond rearrangement [59,60]. The benefits of AFM include a more homogenous polymer network, increased DC, decreased shrinkage, stress, and increased toughness [59]. It is assumed that the inclusion of AFM is responsible for the increased DC, leaving fewer monomers unreacted. Furthermore, aside from aliphatic UDMA, aromatic UDMA is also a component of FOB. The proximity of reactive groups facilitates the reaction diffusion, which may lead to an increased DC, and the planar geometry of benzene rings allows for the building of tighter structures [22]. The amount of free residual monomer, however, does not necessarily correlate with the DC, since the carbon–carbon double bonds may remain as pendant groups bonded to the polymer structure and are not free to be released [22]. It is worth mentioning, as a limitation of this study, that the DC was not measured.

FZ composition differs from FOB; nevertheless, the monomer release was similarly low. The copolymerization of BisGMA and TEGDMA with UDMA and BisEMA may result in synergistic effects on double bond conversion and rotational freedom, thus increasing polymer network homogeneity [22].

Supporting the above findings, the results of the general linear model and the partial eta-squared statistics proved, that both *Material* and *Temperature* as influencing factors, as well as their interaction, have a strong effect on monomer elution.

## 4. Materials and Methods

### 4.1. Sample Preparation

During this in vitro study, six brands of high-viscosity RBC were analyzed. The brands, the manufacturers, the acronym codes, and the chemical compositions are presented in Table 3.

According to the pre-polymerization temperature of the RBC, specimens were divided into two experimental groups. The pre-polymerization temperature of the RBC in the first group was 24 ± 1 °C (room temperature—RT), while RBCs in the second group were pre-heated (PH) before the sample preparation. The thermoviscous VCB was pre-heated by VisCalor Dispenser (VOCO, Cuxhaven, Germany) using the T1 setting (30 s pre-warming to 65 °C). This pre-heating dispenser uses near-infrared technology for rapid warming and provides immediate application with the same device. The pre-warming of the other RBCs was undertaken by the Ena Heat Composite Heating Conditioner (Micerium, Avegno, Italy) using the T2 setting (55 min pre-warming of the device to 55 °C and 15 min pre-warming of the RBC). Five specimens were prepared in each group from each material. The specimens were prepared in a cylindrical polytetrafluoroethylene (PTFE) mold with an internal diameter of 6 mm, an external diameter of 12 mm, and a height of 4 mm. The mold was placed on a thermostatically controlled (30 ± 1 °C) glass slide to represent the isolated tooth. The bulk-fill materials (VCB and FOB) were used in a 4 mm-thick bulk layer; meanwhile, the contemporary RBCs were stratified in 2 × 2 mm layers. A room-temperature hand instrument was used to perform the condensation of the RBCs. Before light-curing, the sample was covered with a polyester strip to avoid contact with oxygen. The 4 mm specimens and both layers of the 2 × 2 mm samples were irradiated with a Light Emitting Diode (LED) curing unit (LED.D, Woodpecker, Guilin, China; average light output given by the manufacturer 850–1000 mW/cm^2^; Λ = 420–480 nm; 8 mm exit diameter fiberglass light guide) in full mode for 20 s, powered by a line cord. The tip of the fiberglass light guide was centrally positioned and parallel to the mold and in direct contact with the polyester strip, which covered the RBC. The irradiance of the LED unit was monitored before and after polymerization with a radiometer (CheckMARC, Bluelight Analytics, Halifax, Canada).

### 4.2. Micro-Computed Tomography Measurements

To analyze the closed-pore volume, micro-computed tomography (micro-CT) scans were performed (Skyscan 1176, Bruker, Kontich, Belgium) on the samples after 24 h following polymerization. Each specimen was scanned for 36 min. The parameters (operating energy: 80 kV, 350 μA; resolution: 8.74 μm/slice; rotation step: 0.7°, exposure time: 1500 ms; and the filter: Al 1 mm) for the micro-CT device were kept constant for all measurements. The SkyScan reconstruction program (NRecon, v.1.7.4.2, Bruker, Kontich, Belgium) was used to reconstruct the raw images and prepare for analysis. Images were converted to 1404 × 1404 pixel resolution in *.bmp format. The 3D microarchitecture analyses of the images were performed according to the following workflow (Skyscan software CTAn, v.1.20.8.0, Bruker, Kontich, Belgium): raw image acquisition, identification of the region of interest, binary selection, morphometry, and custom processing. The region of interest (ROI) included the entire RBC specimen. The pores were calculated using the grayscale images processed with a Gaussian low-pass filter for noise reduction. A global threshold was used to process the gray level ranges to obtain an imposed image of only black and white pixels. The volume of internal voids relative to the total volume of the RBC samples was calculated (%) by measuring the internal voids and specimen volumes of each RBC specimen.

### 4.3. Reversed-Phase High-Performance Liquid Chromatography Measurements

The specimens were kept in separate glass vials, fully immersed in 1.0 mL of 75% ethanol/water storage medium, and stored in a 37 °C incubator for 72 h. The qualitative and quantitative analysis of the eluted unreacted monomers was performed from the collected medium. Reversed-phase high-performance liquid chromatography (RP-HPLC) measurement was used for the analysis. The RP-HPLC system (Dionex Ultimate 3000, Thermo Fisher Scientific Inc., Sunnyvale, CA, USA) consists of the following units: Dionex LPG 3400 SD gradient pump, Rheodyne injector (Rheodyne, CA, USA), and a Dionex DAD 3000 RS UV-VIS detector (Dionex GmbH, Germering, Germany). Chromeleon software (version: 7.2.10) was used to collect the data. The separations were performed on a Brisa “LC^2^” (Teknokroma, Sant Cugat del Vallés, Spain) C18 reversed-phase column (250 mm × 4.60 mm; particle size: 5 μm) and a general reversed/apolar stationary phase C18 with gradient elution. The more polar mobile phase, such as 100% bidistilled water, was used as eluent “A”, whereas mobile phase “B” was 100% *v*/*v* acetonitrile (ACN) (VWR International, Radnor, PA, USA). ACN as the mobile phase is advantageous because of its high elution force and relatively low viscosity. Furthermore, it has lower self-absorption at the detection wavelength used to detect the eluted monomers. During the 30 min chromatographic separation procedure, the “B” eluent content was increased from 40% to 95%. The flow rate was 1.2 mL × min^−1^. The content of mobile phase B was decreased from 95% to 40% in 1 min as the regeneration of the stationary phase (31–46 min), and afterward, the system was washed with 40% “A”. Since the polarity of the monomers varies, but not in such a wide range to reach the complete separation of the components, the polarity of the mobile phase is continuously changed by varying its composition during the chromatographic measurement.

Wavelengths of 205, 215, 227, and 254 nm were tested to detect the eluted monomers.

The evaluation relied on the data collected at a 205 nm wavelength, which was found to be optimal for monomer detection. The amounts of the eluted monomers BisGMA, UDMA, TEGDMA, DDMA, and TCDDD (*Merck KGaA*, Darmstadt, Germany) were calculated using the calibration curve with the areas under the curve of peaks produced by the monomers, respectively. The amounts of released monomers were calculated for every 1 mg of RBC. TEGDMA, UDMA, BisGMA, TCDDD, and DDMA standard monomers had retention times of 11.07, 16.10, 18.42, 23.39, and 29.20 min, respectively. The peaks were well separated from each other.

### 4.4. Statistical Analysis

Previous study results [26] and sample size formula were used to estimate sample size [61]. Sample size formula:

$$n=\frac{{\left(z_{1-\frac{\alpha }{2}}+z_{1-\beta }\right)}^{2}{\left(s_{1}+s_{2}\right)}^{2}}{{\left(M_{1}-M_{2}\right)}^{2}}$$

where z = standard score; α = probability of Type I error at 95% confidence level = 0.05; z_1−α/2_ = 1.96 for 95% confidence; β = probability of Type II error = 0.20; 1 − β = the power of the test = 0.80; z_1−β_ = value of standard normal variate corresponding to 0.80 value of power = 0.84; s_1_ = standard deviation of the outcome variable of group 1 = 0.01; s_2_ = standard deviation of the outcome variable of group 2 = 0.08; M_1_ = mean of the outcome variable of group 1 = 0.22; and M_2_ = mean of the outcome variable of group 2 = 0.08. The predicted sample size (n) was found to be a total of 3.24 samples per group. According to the calculation, n = 5 per group sample size was selected.

The statistical analyses were performed with SPSS (Version 26.0; IBM, Armonk, NY, USA). The Kolmogorov–Smirnov test was applied to test the normal distribution of the data, followed by a parametric statistical test.

The closed-porosity volume of the investigated RBCs was compared with a one-way analysis of variance (ANOVA). Tukey’s post hoc adjustment was used for multiple comparisons.

A two-tailed independent *t*-test was applied to compare the difference in porosity and monomer elution between the room temperature and pre-heated groups of the investigated RBCs.

The general linear model and partial eta-squared statistics were used to test the influence and describe the relative effect size for *Material* and pre-cure *Temperature* as independent factors. *p*-values below 0.05 were considered statistically significant.

## 5. Conclusions

The elevated pre-polymerization temperature of the investigated bulk-fill and conventional high-viscosity dental RBCs significantly increased the closed-porosity volume relative to the total volume of the RBC samples. The evaluated monomers from the investigated bulk-fill RBCs showed a significantly greater degree of elution when applied at room temperature compared to the pre-heated samples. In contrast, the elution of monomers increased from layered conventional RBCs, when their pre-polymerization temperature was elevated. The absolute amount of released monomers was strongly dependent on the material’s composition and not related to the bulk or layered application method.

As a future direction, a long-term clinical trial is necessary to clarify the effect of pre-heating on the success and survival of RBCs.

## Figures and Tables

**Table 1:** Differences in mean values of eluted monomers (nmol/1 mg RBC) between room-temperature (RT) and pre-heated (PH) resin-based composites. Data are provided using a two-tailed independent *t*-test.

RBC	Monomer	Temp	Mean (S.D.)	t-Value	*p*-Value	95% CI	
						Lower Value	Upper Value
Enamel Plus HRi	UDMA	RT	3.5 (0.13)	26.5	<0.001	1.37	1.66
		PH	5.02 (0.05)				
	DDMA	RT	4.89 (0.24)	10.1	<0.001	0.005	0.03
		PH	7.57 (0.24)				
	TCDDD	RT	0.05 (0.01)	19.1	<0.001	2.36	3
		PH	0.07 (0.01)				
Estelite Ʃ Quick	TEGDMA	RT	3.44 (1.13)	11.2	<0.001	−0.6	1.78
		PH	4.05 (0.1)				
	BisGMA	RT	4.06 (0.24)	7.9	<0.001	0.79	1.32
		PH	5.12 (0.09)				
Filtek Z250	TEGDMA	RT	0.07 (0.01)	3.7	0.006	−0.003	0.02
		PH	0.06 (0.01)				
	UDMA	RT	0.62 (0.01)	9.6	<0.001	0.19	0.28
		PH	0.86 (0.04)				
	BisGMA	RT	0.27 (0.01)	15.2	<0.001	0.08	0.1
		PH	0.36 (0.01)				
Gaenial Posterior	UDMA	RT	7.81 (0.3)	27.7	<0.001	3.66	4.57
		PH	11.93 (0.3)				
Viscalor Bulk	TEGDMA	RT	0.53 (0.01)	6.7	<0.001	0.05	0.09
		PH	0.45 (0.02)				
	BisGMA	RT	8.6 (0.39)	10.5	<0.001	1.54	2.39
		PH	6.6 (0.14)				
	DDMA	RT	0.1 (0)	1.5	0.17	−0.003	0.02
		PH	0.1 (0)				
Filtek One Bulk	UDMA	RT	0.49 (0.02)	2.7	0.28	0.004	0.05
		PH	0.46 (0.01)				
	BisGMA	RT	0.59 (0.02)	2.5	0.04	0.003	0.07
		PH	0.55 (0.03)				
	DDMA	RT	1.21 (0.04)	3.9	0.004	0.03	0.13
		PH	1.13 (0.03)				

Abbreviations: RBC: resin-based composite; Temp: temperature; S.D.: standard deviation; CI: confidence interval; BisGMA: bisphenol-A diglycidil ether dimethacrylate; DDMA: 1,12-dodecane dimethacrylate; TCDDD: tricyclodecane dimethanol dimethacrylate; TEGDMA: trietylene glycol di-methacrylate; UDMA: urethane dimethacrylate.

**Table 2:** Relative effect size of *Material* (*M*) and *Temperature* (*T*) factors and their interaction (*M* × *T*) on the amounts of released monomers. The general linear model and partial eta-squared statistics (η^2^) show a large and significant effect of both the type of resin composite (*M*) and pre-polymerization temperature (*T*) and their interaction on the release of those monomers that can be found in more resin composites.

Factor	Monomer release							
	BisGMA		UDMA		TEGDMA		DDDMA	
	*p*-Value	η^2^	*p*-Value	η^2^	*p*-Value	η^2^	*p*-Value	η^2^
*Material*	<0.001	0.99	<0.001	0.99	<0.001	0.99	<0.001	0.99
*Temperature*	<0.001	0.98	<0.001	0.98	<0.001	0.79	<0.001	0.93
*M × T*	<0.001	0.98	<0.001	0.98	<0.001	0.92	<0.001	0.97

Abbreviations: BisGMA: bisphenol-A diglycidil ether dimethacrylate; DDDMA: 1,12-dodecane dimethacrylate; TEGDMA: trietylene glycol di-methacrylate; UDMA: urethane dimethacrylate.

**Table 3:** Composition, manufacturers, codes, and pre-polymerization temperature of the investigated resin-based composites.

Material	Manufacturer	PPT	Acronym Code	Matrix	Filler	Filler Load
VisCalor Bulk	Voco, Cuxhaven, Germany	24 °C	VCB_24	BisGMA, aliphatic DMA	Nano-hybrid (not detailed by the company)	83 wt%
		65 °C	VCB_65			
Filtek One Bulk Fill Restorative	3M ESPE, St. Paul, MN, USA	24 °C	FOB_24	AFM, UDMA, AUDMA, DDMA	20 nm silica, 4–11 nm Zr, Zr-silica, 0.1 µm ytterbium-trifluoride	58.5 vol% 76.5 wt%
		55 °C	FOB_55			
Filtek Z250	3M ESPE, St. Paul, MN, USA	24 °C	FZ_24	BisGMA, BisEMA, TEGDMA, UDMA	0.01–3.5 µm (mean 0.6 µm) silanated Zr-silica	60 vol% 82 wt%
		55 °C	FZ_55			
G-ænial Posterior	GC, Leuven, Belgium	24 °C	GP_24	UDMA, TCDDD DMA	F-Al-silicate, Sr-glass, lanthanide-F	65 vol% 77 wt%
		55 °C	GP_55			
Enamel Plus Hri Bio Function	Micerium S.p.A., Avegno, Italy	24 °C	EP_24	UDMA, TCDDD	0.005–0.05 µm dispersed SiO_2_, 0.2–3 µm glass particle	60 vol% 74 wt%
		55 °C	EP_55			
Estelite Sigma Quick	Tokuyama, Tokio, Japan	24 °C	ESQ_24	TEGDMA, BisGMA	0.2 µm spherical Si-Zr, TiO_2_	71 vol% 82 wt%
		55 °C	ESQ_55			

Abbreviations: PPT: pre-polymerization temperature; BisGMA: bisphenol-A diglycidil ether dimethacrylate; DMA: dimethacrylate; AFM: addition fragmentation monomer; UDMA: urethane dimethacrylate; AUDMA: aromatic urethane dimethacrylate; 1,12-DDMA: 1,12-dodecane dimethacrylate; BisEMA: bisphenol A ethoxylate dimethacrylate; DUDMA: diurethane dimethacrylate; TEGDMA: trietylene glycol dimethacrylate; TCDDD: tricyclodecane dimethanol dimethacrylate; BDDMA: 1,4-butanediol dimethacrylate; vol%: volume%; wt%: weight%.

## Data Availability

Data are available from the corresponding author.
